# Impact of Doxorubicin Treatment on the Physiological Functions of White Adipose Tissue

**DOI:** 10.1371/journal.pone.0151548

**Published:** 2016-03-25

**Authors:** Luana Amorim Biondo, Edson Alves Lima Junior, Camila Oliveira Souza, Maysa Mariana Cruz, Roberta D. C. Cunha, Maria Isabel Alonso-Vale, Lila Missae Oyama, Claudia M. Oller Nascimento, Gustavo Duarte Pimentel, Ronaldo V. T. dos Santos, Fabio Santos Lira, José Cesar Rosa Neto

**Affiliations:** 1 Immunometabolism Research Group, Department of Cellular Biology and Development, Institute of Biomedical Sciences, University of São Paulo (USP), São Paulo, SP, Brazil; 2 Department of Biological Sciences, Institute of Environmental Sciences, Chemical and Pharmaceutical, Federal University of Sao Paulo (UNIFESP), São Paulo, SP, Brazil; 3 Department of Physiology, Physiology of Nutrition Discipline, Federal University of São Paulo (UNIFESP), São Paulo, SP, Brazil; 4 Faculty of Nursing and Nutrition, Federal University of Goiás (UFG), Goiânia, GO, Brazil; 5 Department of Psychobiology, Universidade Federal de São Paulo—UNIFESP, São Paulo, SP, Brazil; 6 Department of Physical Education, State University of São Paulo "Júlio de Mesquita Filho" (UNESP), Presidente Prudente, SP, Brazil; East Tennessee State University, UNITED STATES

## Abstract

White adipose tissue (WAT) plays a fundamental role in maintaining energy balance and important endocrine functions. The loss of WAT modifies adipokine secretion and disrupts homeostasis, potentially leading to severe metabolic effects and a reduced quality of life. Doxorubicin is a chemotherapeutic agent used clinically because of its good effectiveness against various types of cancer. However, doxorubicin has deleterious effects in many healthy tissues, including WAT, liver, and skeletal and cardiac muscles. Our objective was to investigate the effects of doxorubicin on white adipocytes through *in vivo* and *in vitro* experiments. Doxorubicin reduced the uptake of glucose by retroperitoneal adipocytes and 3T3-L1 cells via the inhibition of AMP-activated protein kinase Thr172 phosphorylation and glucose transporter 4 content. Doxorubicin also reduced the serum level of adiponectin and, to a greater extent, the expression of genes encoding lipogenic (*Fas* and *Acc*) and adipogenic factors (*Pparg*, *C/ebpa*, and *Srebp1*c) in retroperitoneal adipose tissue. In addition, doxorubicin inhibited both lipogenesis and lipolysis and reduced the hormone-sensitive lipase and adipose tissue triacylglycerol lipase protein levels. Therefore, our results demonstrate the impact of doxorubicin on WAT. These results are important to understand some side effects observed in patients receiving chemotherapy and should encourage new adjuvant treatments that aim to inhibit these side effects.

## Introduction

White adipose tissue (WAT) is a complex organ, that is composed by mature white adipocytes, approximately between one to two thirds of total WAT cells [1]. The other type of cells that make up the adipose tissue are fibroblasts, macrophages, pre adipocytes and endothelial cells [2]. This tissue has a high capacity for remodeling in response to different stress, such as hypoxia, loss or gain of mass, and excess or deprivation of nutrients [3].

The WAT is the major energy reservoir in animals [2]. Moreover, this tissue regulates several metabolic processes that are necessary for maintaining energetic homeostasis. WAT is responsible for approximately 15% of all glucose metabolism, and this metabolic substrate is utilized for both energy production and storage in adipocytes [4–5]. In addition, the storage, mobilization and supply of fatty acids are regulated by WAT. The process of storage is termed lipogenesis, which is a complex metabolic route to fatty acid synthesis (mainly from glucose) and storage; and on the other hand lipolysis is a route for the mobilization and supply of free fatty acids by other organs [1, 4, 6].

Finally, the WAT mass is also influenced by adipogenesis, which is a process that leads to differentiation of the preadipocytes in mature adipocytes. This process is managed by several hormones (insulin; corticosterone and other growth factors) and a complex activation in a time-dependent manner of diverse transcription factors (*C/EBPa*,*b*,*g; SREBP-1c* and *PPARg* encodes CCAAT enhancer binding protein alpha, beta, gamma; Sterol-regulatory-element-binding protein 1c and Peroxisome-proliferator-activated receptor gamma) [7–8].

The WAT cross talks with different organs in the body, and since 1994 its role as an endocrine organ was established; since it produces and releases different adipokines [9]. The excessive gain or loss of WAT modifies the secretion of adipokines and cytokines, and can thus alter appetite, reproduction, circadian rhythms, energy expenditures, and inflammation, resulting in a reduced quality of life [9–10]

The chemotherapeutic agent doxorubicin was developed in the 1960s, and it is currently used widely in clinical practice because of its strong antitumor activity. This agent exhibits poor tumor selectivity and is therefore used to treat a variety of malignancies, including breast cancer, lymphoblastic leukemia, breast carcinoma, ovarian carcinoma, and both Hodgkin and non-Hodgkin lymphomas [11–12]. Patients undergoing doxorubicin chemotherapy commonly develop symptoms such as vomiting, diarrhea, irregular heartbeat, mouth sores, abnormal bleeding, and swelling of the feet or ankles [13–14]. Furthermore, doxorubicin treatment is characterized by deleterious side effects in healthy tissues, including sarcopenia, diabetes, nephrotoxicity [15–16], hepatotoxicity [17], and severe cardiocytotoxicity, according to reports as early as 1974 [18].

The other chemoterapic drug, which also possesses a large spectrum, is cisplatin, leads to an impairment of lipid metabolism, with an increase in lipolysis, and an inhibition of lipogenesis and adipogenesis due to loss of WAT and body weight [19] in mice without tumors. This phenomenon is very similar with a cachexia syndrome (cachexia-like syndrome),which is a complex metabolic disorder in which patients exhibit weight loss and depletion of adipose tissue and skeletal muscle [19]; in addition, severe symptoms such as anorexia and extreme fatigue may occur in patients with cancer. These effects lead to poor survival rates and elevated toxicity associated with treatment [14].

In accordance with metabolic disorders caused by doxorubicin treatment, data collected in our laboratory have demonstrated that doxorubicin can induce hyperglycemia, decrease insulin sensitivity, and increase levels of nonesterified fatty acids. Circulating glucose uptake occurs in the skeletal muscle and adipose tissue in response to insulin stimuli. Recently, we demonstrated that AMP-activated protein kinase (AMPK) and glucose transporter 4 (GLUT-4) signaling in skeletal muscle is disrupted by doxorubicin treatment [20].

Based on this characterization of metabolic disturbances after doxorubicin treatment, the aim of this study was to investigate the effects of doxorubicin on WAT at the cellular level through *in vivo* and *in vitro* investigations of processes such as lipolysis, lipogenesis, glucose uptake, and adipogenesis.

## Results

### Doxorubicin promotes toxicity in 3T3L1 cells

**Fig 1:**
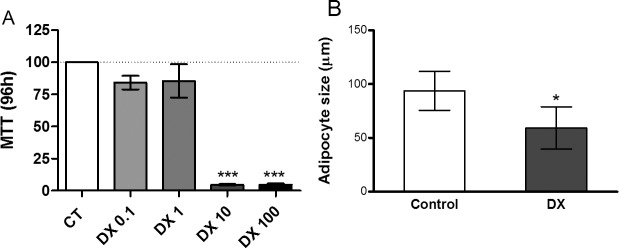
Doxorubicin promotes toxicity in 3T3L1 cell. Cell viability after 96 hours (A) of doxorubicin (DX) incubation. Concentrations range from 0.001 to 100 uM. Cell viability was assessed by MTT assay and normalized adopting the control absorbance as 100% survival. Values represent the mean ± standard deviation of 3 independent experiments. Adipocyte size of cells from the retroperitoneal adipose tissues (B) of rats treated with a single dose of doxorubicin (15 mg/kg body weight ip), euthanized 72 hours after administration. Values represent the mean ± standard deviation of 5 to 6 experiments. The groups were compared using the one-Way ANOVA followed by Dunnett's (A) and t test (B).*p <0,05, **p <0.01, *** p <0.001.

We initially observed that doxorubicin caused considerable lethality in 3T3-L1 cells after 96-h incubation and was nontoxic only at doses <1μM (Fig 1A); similarly, intraperitoneal (i.p.) doxorubicin administration [15 mg/kg body weight (b.w.)] promoted a reduction in adipocyte size in Wistar rats (Fig 1B).

### Doxorubicin reduced glucose uptake

**Fig 2:**
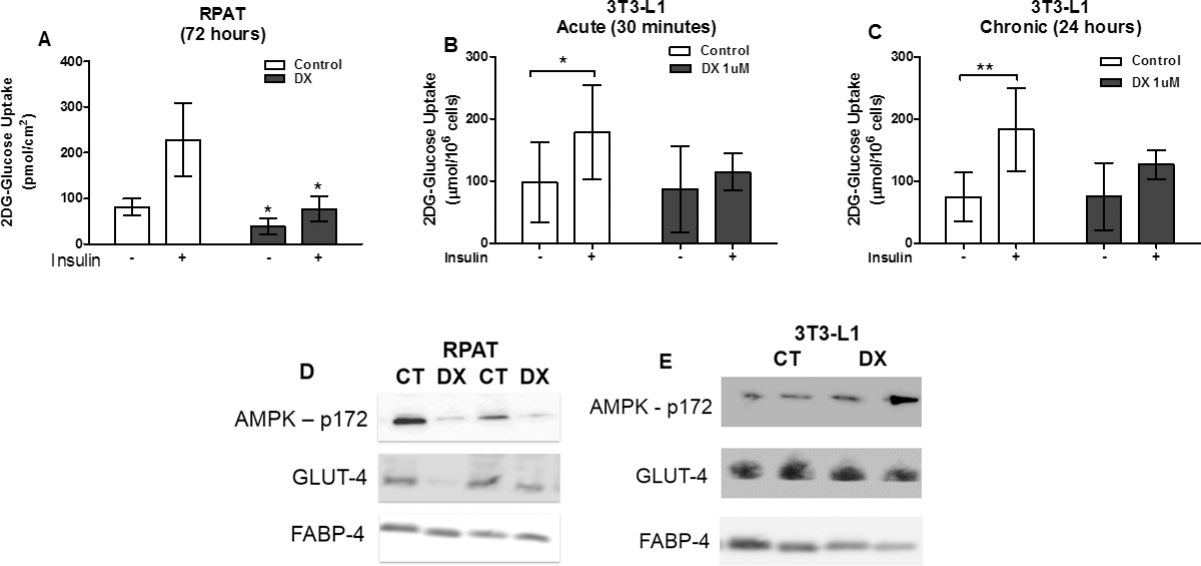
Reduction of glucose uptake by doxorubicin in adipocytes. Glucose uptake from the retroperitoneal adipose tissues of rats (A) and glucose uptake from 3T3L1 cells (B and C). Protein expression of AMPK thr 172 phosphorylated, total content of GLUT4 and FABP4 from retroperitoneal adipose tissues of rats (D) and protein expression of AMPK threonine 172 phosphorylated, total content of GLUT4 and GAPDH from 3T3L1 cells (E). Adipocytes from the retroperitoneal adipose tissues of rats treated with a single dose of doxorubicin (15 mg/kg body weight ip), euthanized 72 hours after administration. The glucose uptake was measured by uptake of 2-DG with and without insulin stimulation (100nMol) in isolated adipocytes and 3T3L1 cells. 3T3L1 cells were incubated with doxorubicin (1uM) for 30 minutes (B) and 24 hours (C and E), after differentiation into mature adipocytes. Values represent the mean ± standard deviation of 5 to 6 experiments (A, B and C). Figures represents 4 experiments per group (D and E). The groups were compared using two-way ANOVA followed by Dunn’s (A, B, and C). *p<0.05 and **p<0.01.

Retroperitoneal adipose tissues from rats treated with doxorubicin (72 h) exhibited reduced glucose uptake after insulin stimulation (Fig 2A). Even at low doses (1uM) and over short periods (30 min or 24 h), doxorubicin treatment reduced glucose uptake after insulin stimulation (Fig 2B and 2C).

### Doxorubicin treatment decreases AMPK phosphorylation and GLUT-4 expression *in vivo*

**Fig 3:**
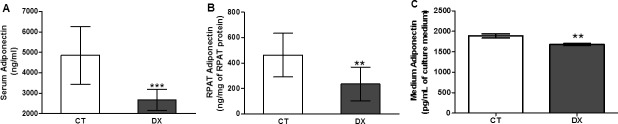
Doxorubicin decreased the adiponectin levels. Adiponectin protein levels in serum (A) and retroperitoneal adipose tissue (B) of rats 72 hours after treatment with doxorubicin (15mg/kg). Concentration of adiponectin released into culture medium (C) with doxorubicin (DX) 1uM for 24 hours, after differentiation into mature adipocytes. Data are mean ± standard deviation of 6 experiments. The groups were compared using the Student t test.** p <0.01, *** p <0.001.

GLUT-4 expression and AMPK Thr172 phosphorylation were reduced in the retroperitoneal adipose tissues of rats treated with doxorubicin (Fig 2D) but not in treated 3T3-L1 cells (Fig 2E). In keeping with the inhibition of AMPK Thr172 phosphorylation, we observed that doxorubicin reduced adiponectin levels in both the sera (Fig 3A) and retroperitoneal adipose tissues (Fig 3B) of rats. Low-dose (1uM) doxorubicin treatment over a 24-h period also suppressed levels of adiponectin secreted into the culture medium (Fig 3C).

### Doxorubicin affects lipogenesis

**Fig 4:**
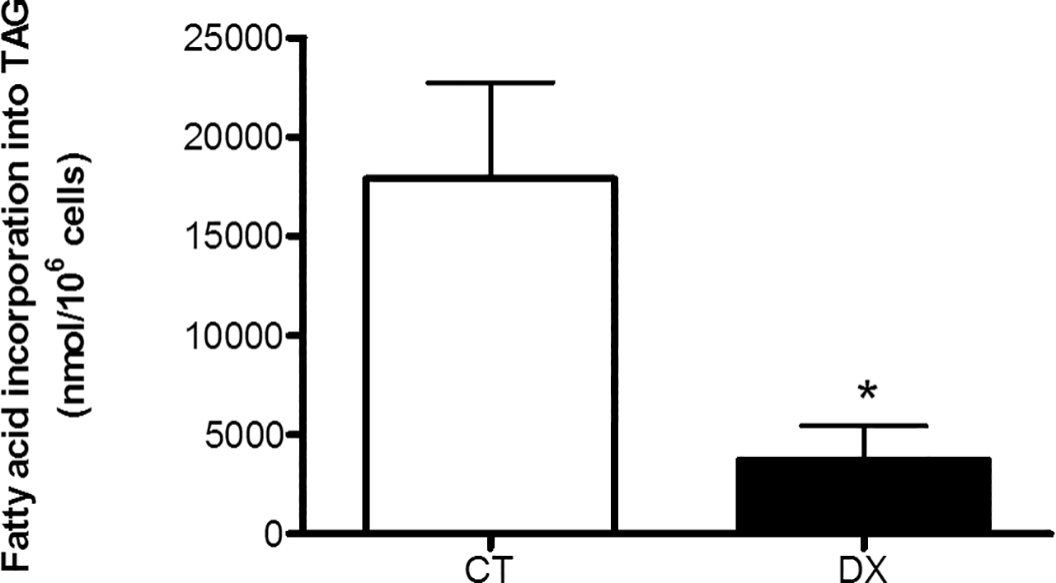
Doxorubicin reduces lipogenesis. Fatty acid incorporation into triacylglycerol (TAG) in isolated adipocytes of retroperitoneal adipose tissue from rats after 72 hours of doxorubicin (DX) (15 mg/kg weight) or saline (Control) injection i.p. Data are mean ± standard deviation of 4 to 6 experiments. The groups were compared using the Student t test, *p<0.05.

**Fig 5:**
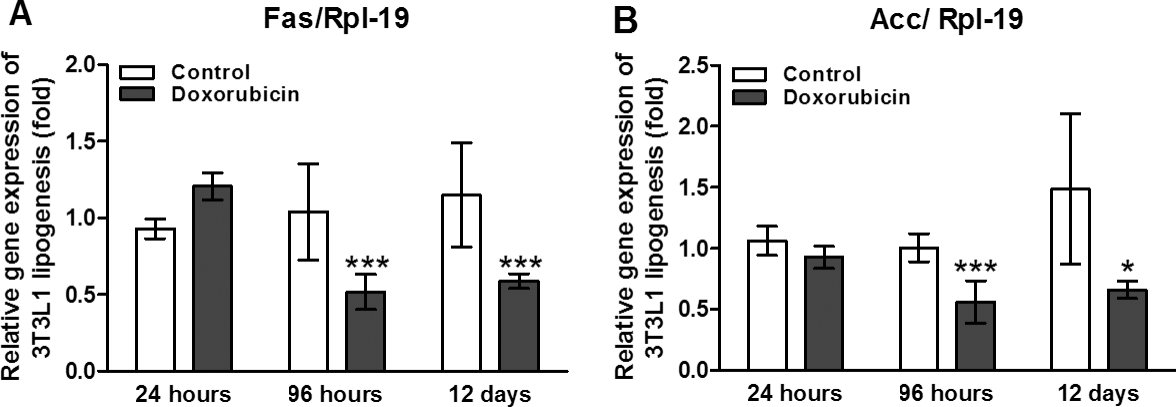
Doxorubicin reduces lipogenic enzymes. Gene expression of lipogenic enzymes *Fas* (A) and *Acc* (B) at 24 hours, 96 hours and 12 days after induction of cell differentiation in 3T3L1 cells treated with 1uM of doxorubicin. The gene expression was evaluated by real time PCR. Data are mean ± standard deviation of 4 to 6 experiments. The groups were compared using the Two way ANOVA followed by Bonferroni post test, *p <0.05, **p<0.01 and ***p<0.001.

**Fig 6:**
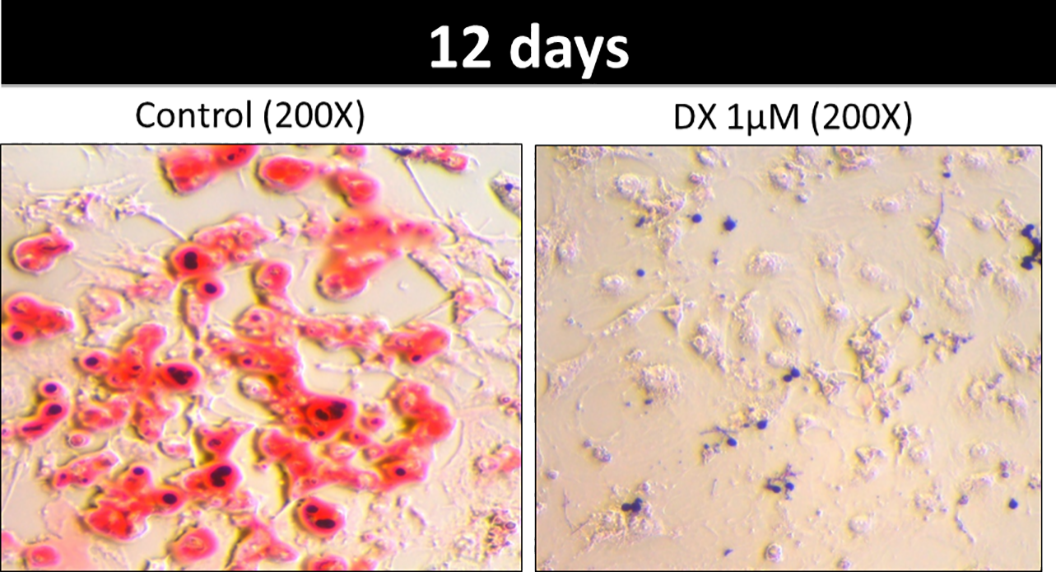
Doxorubicin impairs lipogenesis. 3T3L1 cells stained with oil red. 3T3L1 cell were treated with doxorubicin (DX) 1uM or PBS (control) for 12 days after the differentiation cocktail. Photos were obtained by optical microscopy at a resolution of 200X.

In addition, doxorubicin treatment compromised several metabolic pathways. Rats injected with doxorubicin exhibited reduced lipid synthesis from palmitate (*ex vivo*; Fig 4). Likewise, 3T3-L1 cells treated with doxorubicin exhibited lower *de novo* expression levels of genes associated with lipogenesis regulation. For instance, both *Fas* (encodes fatty acid synthase; Fig 5A) and *Acc* (encodes acetyl-CoA carboxylase; Fig 5B) expression were inhibited in 3T3-L1 cells from 96 h to 12 days after the induction of differentiation and doxorubicin treatment (Fig 6).

### Adipogenesisis compromised by doxorubicin

**Fig 7:**
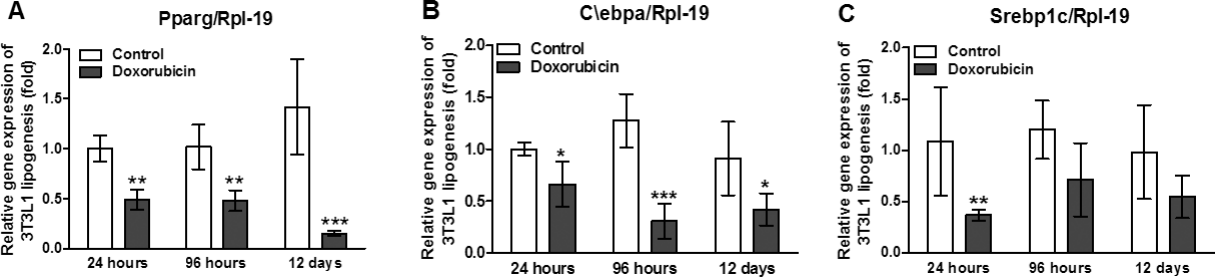
Adipogenesis is compromised by doxorubicin in 3T3L1 cells. Gene expression of adipogenics factors *Ppparg* (A), *C/ebpa* (B) and *Srebp1c* (C) at 24 hours, 96 hours and 12 days after induction of cell differentiation in 3T3L1 cells treated with 1uM of doxorubicin. The gene expression was evaluated by real time PCR. Data are mean ± standard deviation of 4 to 6 experiments. The groups were compared using the Two way ANOVA followed by Bonferroni post test, *p<0.05, **p<0.01 and ***p<0.001.

Adipogenesis was also inhibited by doxorubicin. Oil red staining showed significant disruption of 3T3-L1 differentiation after a 12-day incubation with doxorubicin (1uM; Fig 7). In addition, doxorubicin decreased the expression of genes associated with adipogenesis modulation; in particular, the expression levels of *Pparg* (Fig 7A) and *C/ebpa* (Fig 7B) were strongly reduced following doxorubicin treatment at all three time points, whereas *Srebp1c* expression was only inhibited after a 24 h incubation with doxorubicin (Fig 7C).

### Doxorubicin modifies the lipolysis process

**Fig 8:**
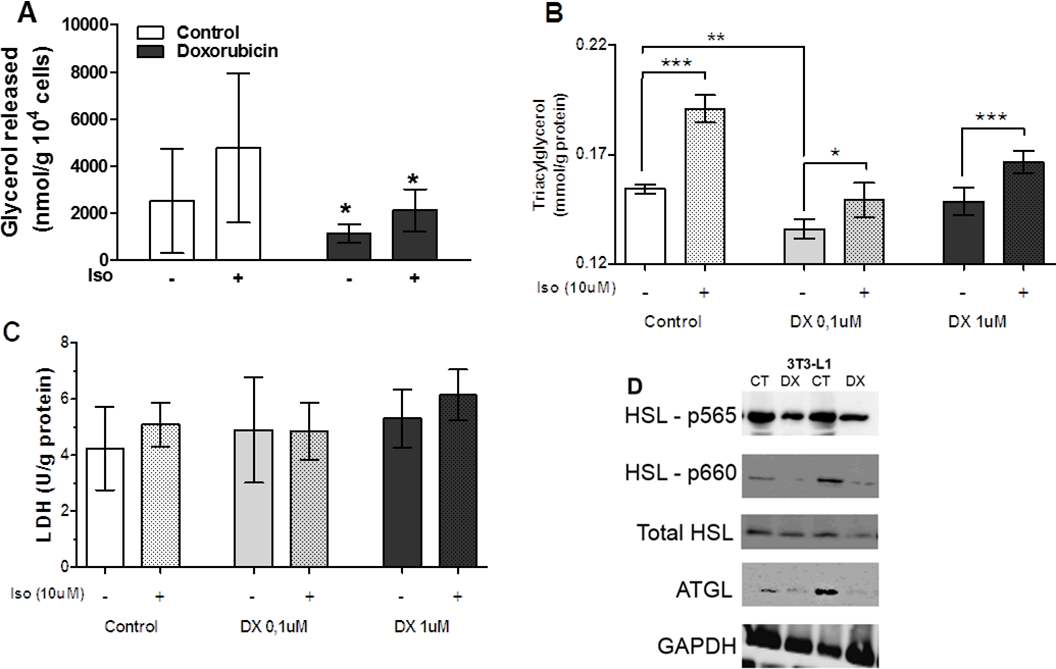
Doxorubicin inhibits the lipolysis process. Glycerol released in assay of lipolysis the adipocytes isolated from retroperitoneal adipose tissue of rats after 72 hours of doxorubicin administration (15mg/kg of weighti.p), with or without isoproterenol stimulation (10uM) for 30 minutes (A). Concentration of glycerol (B) and lactate dehydrogenase LDH in incubation medium of 3T3L1 (C). Lipolysis was stimulated with isoproterenol (Iso) 10uM with doxorubicin (DX) at concentrations of 0.1 and 1uM for 24 hours, the parameters are normalized by the concentration of protein extracted from the cells. Values represent the mean ± standard deviation of 4 to 8 experiments. The groups were compared using the One-Way ANOVA followed by Bonferroni post test. *p<0.05, **p<0.01, ***p<0.001 (A, B and C). Protein expression of HSL phosphorylated at residue Ser-565, Ser-660, total HSL, ATGL and GAPDH (D). Proteins extracted from retroperitoneal adipose tissue of rats 72 hours after treatment with doxorubicin (15mg/kg). Figure represents 4 experiments per group (D).

Finally, we determined that lipolysis was also inhibited by doxorubicin. Although we expected an increase in activity in this pathway, we observed that doxorubicin reduced both basal and isoproterenol-stimulated lipolysis in isolated retroperitoneal cells (Fig 8A). Whereas two different doses of doxorubicin (0.1 and 1uM) failed to induce death in 3T3L1 cells (Fig 8C), both doses reduced lipolysis in both the presence and absence of isoproterenol stimulation (Fig 8B). Similarly, the protein levels of hormone-sensitive lipase (HSL) p565 and p660, which are phosphorylated in retroperitoneal adipose tissue, were reduced by doxorubicin treatment, as was the protein level of adipose tissue triacylglycerol lipase (ATGL; Fig 8D).

## Methods

### Animals

The Experimental Research Committee of the University of São Paulo approved all procedures for the care of the animals used in this study. All experiments were performed in accordance with the approved guidelines of animal ethic committee the ICB-USP, registered under n°5, fls 15, book 03 of this Institute.

26 male Wistar rats approximately 14 weeks of age (weighing 350–380 g), obtained from Biomedical Sciences Institute of University of São Paulo, were housed four per cage in an animal room under 12:12-h light-dark cycle (lights on at 06:00). Rats were randomly divided into two groups: saline control (CT) (n = 13); and the doxorubicin group (DX) (n = 13). After the acclimation period, the DX received 15mg/kg, i.p., doxorubicin chloridrate (Eurofarma Laboratory, Campinas, Brazil); the CT animals received an equal volume of saline.

Rats received food (chow pellet diet; Nuvilab CR1, Nuvital SA, Colombo, PR, Brazil) and water *ad libitum*. Food intake and body weight were assessed daily. Furthermore, after injection with doxorubicin, the health and well-being of the rats were monitored daily.

Rats were euthanized by decapitation 72 hours after the doxorubicin treatment, after which the retroperitoneal adipose tissue was removed, weighed, flash frozen in liquid nitrogen and stored at -80°C. Whole blood was drawn and centrifuged, and serum was removed and kept frozen at –80°C for later analysis.

### Adipocyte isolation

For adipocyte isolation, retroperitoneal fat pads from rats were dissolved in Dulbeccos modified Eagle Medium (DMEM) (Sigma, St. Louis, USA) supplemented with HEPES (20 mM), sodium pyruvate (2 mM), bovine serum albumin (BSA, 1%), and collagenase type II (1 mg/mL), in an orbital bath shaker with a pH of 7.4 and at 37°C. Isolated adipocytes were filtered and washed three times in the same buffer without collagenase. The adipocytes were photographed under an optical microscope (×100 magnification) using a microscope camera (Moticam 1000; Motic, Richmond, British Columbia, Canada), and the adipocyte diameter average was determined by measuring 50 cells using Motic-Images Plus 2.0 software.

### Cell culture

3T3-L1 cells were from ATCC (Manassas, USA)cells between 5–10 passages were plated(2x10^4^) in 24-well, 12-well or 6-well plates, maintained in Dulbecco’s modified Eagle medium (Sigma, St. Louis, USA) at 37°C, 5% CO_2_ and supplemented with 10% fetal bovine serum (FBS) and 3% antibiotic solution (penicillin/streptomycin). The cells were allowed to reach confluence and after two days, a differentiation medium containing 10% FBS, 3% antibiotic, IBMX (0.5mM), insulin (1.6uM) and dexamethasone (1uM) was added to the plates for 2–4 days. Then, the cells were maintained in a modified DMEM feeding containing 10% FBS, 3% antibiotic and insulin (0.4uM) until the 11^th^ day, with medium exchanges every 2^nd^ day. On the 11^th^day cells were maintained in a DMEM feeding with 0.5% FBS for 24 hours and the experimental procedures performed.

### MTT Assay

For the assessment of cell viability, differentiated 3T3-L1 cells were incubated with different concentrations of doxorubicin (0.001 to 100uMol) for 72 and 96 hours in differentiation medium. After these periods, the cell medium was replaced by PBS containing 0.05 mg/mL of MTT (3-(4,5-dimethylthiazol-2-yl)-2,5-diphenyltetrazolium bromide)(Solon, OH, USA) and cultivated for another 3 hours in the cells’ incubator. Supernatant was removed and isopropanol/HCl (11 M) was added, the absorbance was measured at 595 nm. The effect of the doxorubicin on cell viability was relativized by control group.

### 2-Deoxy-D-glucose (2-DG) uptake

Differentiated 3T3-L1 cells (~8×10^5^ cells/well) or primary retroperitoneal adipocytes (10^6^ cells/mL) were washed in PBS and incubated with or without insulin (100 nmol/L) in a buffer composed of (mM): 140 NaCl, 20 Hepes, 5 KCl, 2.5 MgSO4, 1 CaCl2, BSA 1% (pH 7.4), for 20 min at 37°C. Subsequently, 2-deoxy-D-[3H]-glucose (0.4 mmol/L, 1850 Bq/tube or well) (Amershan Bioscience, UK) was added and the reaction was allowed to occur for exactly 4 and 3 min, for 3T3-L1 and primary adipocytes, respectively. The reaction was interrupted by adding 250-μl ice-cold phloretin (0.3mmol/L in Earle's salts, HEPES 10mm, BSA 1% and DMSO 0.05%). The 3T3-L1 cells were washed with cold PBS, 300uLNaOH 50mM was added to each well, the plate was rotated for 20 min and 250uL was collected to measure the radioactivity (1450 LSC, CouterMicroBeta, Trilux, PerkinElmer). For retroperitoneal primary adipocytes, 200-ul doses of this final mixture were layered with 200ul of silicone oil (density of 0.963 mg/ml) in microfuge tubes and centrifuged for 10 sec at 11,000g. The cell pellet on top of the oil layer was collected, transferred to vials containing the scintillation cocktail for radioactivity measurement by a beta counter. The results are expressed as umol of glucose per 1x10^6^ cells and pmol per cm^2^, for 3T3-L1 and retroperitoneal primary adipocytes, respectively.

### Protein analysis by Western Blotting

Retroperitoneal adipose tissues and 3T3-L1 was removed, then homogenized in extraction buffer containing protease and phosphatase inhibitors. Extracts were centrifuged and protein determination in the supernatants was performed by the Bradford assay (Bio-Rad Laboratories, Hercules, CA, USA). The proteins were treated with a Laemmli sample buffer containing dithiothreitol and boiled for 5 min before being loaded into a 10% SDS-PAGE in a Bio-Rad miniature slab gel apparatus. The electrotransfer of proteins from the gel to nitrocellulose was performed. The nitrocellulose membranes were incubate overnight at 4°C with antibodies against GLUT-4, ATGL, phospho AMPK-T172, phospho HSL-S565, HSL-S660, total HSL and FABP4 obtained from Cell Signaling Technology® (Danvers, MA, USA), and GAPDH obtained from Santa Cruz Biotechnology (Santa Cruz, CA, USA). Afterwards, the blots were incubated with a peroxidase-conjugated secondary antibody for (1:5000) 1h. Specific bands were detected by chemoluminescence and visualization/capture was performed by exposure of the membranes to RX films. Band intensities were quantified by optical densitometry of developed autoradiographs (Scion Image software-Scion Corporation, Frederick, Md., USA).

### Adipose tissue and serum adiponectin

Frozen tissues (0.1g) were homogenized in the RIPA buffer (0.625% Nonidet P-40, 0.625% sodium deoxycholate,6.25mM sodium phosphate, and 1 mMethylenediaminetetraacetic acid at pH 7.4) containing 10ug/ml of protease inhibitor cocktail (Sigma-Aldrich, St. Louis, Missouri). Homogenates were centrifuged, the supernatant was saved, and protein concentration was determined using the Bradford assay (Bio-Rad, Hercules, California) with bovine serum albumin as reference. The quantitative assessment of adiponectin in retroperitoneal adipose tissue and serum were carried out by ELISA (DuoSet ELISA, R&D Systems, Minneapolis, MN). All samples were run as duplicates, and mean value was reported.

### Quantitative Real-Time PCR

Total RNA from 3T3L1 cells was extracted with the reagent Trizol (Invitrogen Life Technologies, Carlsbad, USA), and reverse transcribed to cDNA using a High-Capacity cDNA kit (Applied Biosystems, Warrington, UK). The RNA analysis by agarose gel electrophoresis are shown in S2 Fig. Lipogenic enzymes—*Fas* and *Acc*—and adipogenic factors–*Pparg*, *C/ebpa* and *Srebp1c*. Gene expression was evaluated by real-time PCR using a Rotor Gene (Qiagen) and SYBR Green as the fluorescent dye. Quantification of gene expression was carried with RPL-19 gene as a control and is shown in S2 Fig. Primer sequences are shown in Table 1.

**Table 1:** Primer sequences.

GENE	5’-FORWARD PRIMER-3’	5’-REVERSE PRIMER-3’
***Rpl-19***	CAATGCCAACTCCCGTCA	GTGTTTTTCCGGCAACGAG
***Fas***	GATTCGGTGTATCCTGCTGTC	CATGCTTTAGCACCTGCTGT
***Acca***	CCAGCAGATTGCCAACATC	ACTTCGGTACCTCTGCACCA
***Pparg***	ATCTTAACTGCCGGATCCAC	CAAACCTGATGGCATTGTGAG
***C/ebpa***	TAGGTTTCTGGGCTTTGTGG	GATGGATCGATTGTGCTTCA
***Srebp1c***	TGGACCACAGAAAGGTGGA	ATGGCCTTGTCAATGGAACT

### Lipogenesis

Primary retroperitoneal adipocytes (10^6^ cells/mL) were incubated in Krebs/Ringer/phosphate buffer (BSA 1%, 2mM glucose, 200uM [1-14C]-Palmitate, 1850 Bq/tube at pH 7.4) for 2 h at 37°C in a water bath. At the end of incubation, the mixture was transferred to 1.5mL tubes containing 400uL of silicone oil and centrifuged for 30s. The cell pellet on the top of the oil layer was transferred to polypropylene tubes containing 2.5mL of Dole's reagent [isopropanol:n-heptane:H2SO4 (4:1:0.25, vol/vol/vol)] for lipid extraction. After addition of n-heptane (1.5mL) and distilled water (1.5mL), the tubes were vortexed and the mixture decanted for 5min. An aliquot of the upper phase was collected into a scintillation vial for the determination of radioactivity trapped into TAG (1450 LSC, CouterMicroBeta, Trilux; PerkinElmer). Results are expressed as nmol of palmitate incorporated into TAG per 1x10^6^ cells/h.

### Oil Red O staining and lipid content quantification

Oil Red O staining (Sigma-Aldrich) assessed cellular lipid content. After 12 days of differentiation, the cells were washed, fixed in 4% paraformaldehyde for 1 h and stained with an Oil Red O working solution for 1 h. After washing 3 times with PBS, the cells were photographed with a light microscope (Olympus, Osaka, Japan). The size and number of lipid droplets were analyzed using Image Pro Plus 5.02.

### Lipolysis

Lipolysis was estimated as the rate of glycerol release in the incubation medium. For this, retroperitoneal adipocytes (1 x 10^6^ cells/ml) were incubated in Krebs/Ringer/phosphate buffer (pH 7.4) containing BSA (20mM) and glucose (5mM) for 30 min at 37°C in the presence or absence of isoproterenol (2uM). The reaction was stopped on ice and media was carefully collected for measurement of glycerol release (Free glycerol determination kit; Sigma).

After the differentiation, 3T3L1 cels were incubated in glucose transporter buffer (1mM NACL, 0.8 mMMgSO_4_7H_2_0,5.36 mMKCl, 0.845mM NaH_2_PO_4_, 1.5mM CaCl_2_H2O, 25mM Hepes, 0,02μM Fenol Red, 1% BSA,5mM glucose) for 30 min at 37°C in the presence or absence of isoproterenol (10uM). The incubation medium was collectedfor measure of glycerol release (Triacylglycerol Determination Kit, Labtest).

Results expressed as nmol of glycerol per 1x10^4^primary adipocytes and mmol per gram for 3T3-L1.

### Statistical Analysis

Statistical analysis was performed using the GraphPad Prism statistics software package version 5.0 for Windows (GraphPad Software, San Diego, CA, USA). The data are expressed as the means ± SD. Data were analyzed using a Student’s t-test for comparison between two groups. Implementation of the Kolmogorov-Smirnov test revealed that the results of experiments were distributed normally. For comparison of assays in cell culture, the ANOVA one-way test or ANOVA two-way test with Bonferroni post-test were used. A value of P < 0.05*, P<0.01**, P<0.001*** was considered statistically significant.

## Discussion

Previous results reported by our group demonstrated that in Wistar rats, the i.p administration of doxorubicin promoted both a considerable weight loss and reduction in epididimal adipose tissue. These results were similar to those found in the literature, where in low (2.5mg/Kg weekly) doses of doxorubicin were reported to cause reductions in body and adipose tissue weights [21–22]. Based on these findings, we proposed to investigate the toxicity of doxorubicin, as well as its potential role in metabolic impairment in adipose tissue, as clinical studies have demonstrated that a decrease in adipose mass is associated with a poor prognosis in cancer patients [23–25]. Here we showed that doxorubicin was toxic in a both models, causing 3T3-L1 cell death, and that doxorubicin administration could impair glucose uptake, lipogenesis, adipogenesis, and lipolysis both *in vivo* (15 mg/kg b.w. in rats) and *in vitro* (1 and 0.1 μMin 3T3-L1 cell culture). From our results, it is clear that doxorubicin treatment disrupt the adipose tissue homeostasis.

Doxorubicin reduced the viability of adipocytes *in vitro* (Fig 1A) and reduced the diameter of adipocytes *ex vivo* (Fig 1B). WAT has an endocrine function; specifically, it produces and releases adipokines that are important to the maintenance of physiological conditions [26] and supplies the fatty acids needed for energy utilization during periods of energy deprivation [27–28].

WAT is an important tissue in the regulation of glucose homeostasis. In recent studies [29], experiments involving subcutaneous adipose tissue transplantation in sedentary mice demonstrated that the transplanted WAT sufficiently attenuated insulin resistance and thus improved glucose tolerance.

In WAT, glucose uptake is stimulated by insulin. The i.p. administration of doxorubicin led to a reduction in glucose uptake (Fig 2). Under physiological conditions, glucose is mainly transported across the cell membrane through stimulation induced by GLUT-4 isoform translocation [30]. We observed that doxorubicin caused a reduction in GLUT-4 protein expression and AMPK Thr172 phosphorylation *in vivo* (Fig 2D). The reduction of glucose uptake observed in a 3T3-L1 cell-based *in vitro* assay was similar to that observed in *ex vivo* WAT (Fig 2A), except that AMPK Thr172 phosphorylation and GLUT-4 expression were not decreased (Fig 2D and 2E).

The reduction in glucose uptake in insulin-stimulated cells might be attributable to the significant (*P*< 0.01) decrease in *Pparg* expression (Fig 7A). The nuclear receptor *PPARg* is strongly expressed in WAT. Activation of this receptor in response to the administration of drugs from the thiazolidinedione family of agents is utilized clinically to increase insulin sensitivity [31–32]. *PPARg* activation was shown to improve glucose uptake in adipocytes isolated from WAT by increasing GLUT-4 protein expression [33]. By the same token, inhibition of PPAR*g* reduced glucose uptake in adipocytes through reductions in GLUT-1 and GLUT-4 expression [34].

PPAR*g* is the master transcription factor in adipose tissue. Beyond its function in glucose homeostasis, this nuclear receptor is responsible for pleiotropic effects such as the modulation of lipogenesis, adipogenesis, and lipolysis [35]. However, the mechanisms by which *PPARg* regulates these different metabolic processes depend on synergic actions with other transcription factors [36–37].

Adiponectin, the most potent endogenous insulin sensitizing agent, is a protective hormone produced by adipocytes and is important for maintaining energy homeostasis. Generally, the plasma concentrations of adiponectin are inversely proportional to the level of insulin resistance [38]. This effect is normally derived from the activation of AMPK and signaling molecules such as the protein kinase RAS-associated protein Rab5, p38 mitogen-activated protein kinase, phosphoinositide-3-kinase, and AKT [39]. However, lower serum adiponectin levels were observed in retroperitoneal adipose tissues and cell culture supernatants after doxorubicin treatment (Fig 3A–3C). It is important to note that Maruyana et al. (2011) found that the administration of adiponectin protected animals against doxorubicin-induced cardiotoxicity via AKT signaling. Taken together, decreases in *PPARg* and adiponectin could be the cause or effect of this pronounced decrease in glucose uptake and homeostasis associated with doxorubicin treatment.

In addition to its effects on glucose uptake, doxorubicin disrupted several other metabolic pathways, leading to a reduction in lipogenesis *in vivo* (Fig 4) that was accompanied by a decrease in the gene expression of two master enzymes involved in *de novo* lipogenesis, *Fas* and *Acc* (Fig 5). The reduced utilization of glucose to form triacylglycerol and the reduction in *Fas* and *Acc* expression contributed to severe adipocyte biological side effects. Moreover, GLUT-4 protein expression was dramatically reduced in the adipose tissues of rats treated with doxorubicin. Similarly, lipogenesis is mainly regulated by insulin in adipose tissue [40].

Doxorubicin treatment impaired adipogenesis (Fig 6), as well as lipogenesis. Notably, doxorubicin reduced the expression of *C/ebpa*, *Pparg*, and *Srebp1c*, which encode key regulators of adipocyte development. C/EBP belongs to a family of transcription factors that regulate other transcription factors, which in turn induce *Pparg* expression. C/EBP also plays a critical role in adipocyte development and, consequently, lipid accumulation or lipogenesis. SREBP1 is another pro-adipogenic factor that appears to contribute to the expression of *Pparg* and *C/ebpb* [41]. In an earlier report, doxorubicin suppressed adipogenesis after a brief exposure (3 h), thus corroborating our findings. This inhibition was dose dependent, and expression of the transcription factors *C/EBPb* and *PPARg* was also suppressed [42].

*Ex vivo*, both basal and stimulated lipolysis activities were reduced, and we also observed a reduction in phosphorylation of the major lipase HSL at 565Ser and 660 Ser, as well as ATGL in the retroperitoneal adipose tissues of rats treated with doxorubicin (Fig 8D). This reduction in lipolysis was observed via a reduction in glycerol release from adipocytes. Lipolysis depends on a cycle between fatty acid re-esterification and hydrolysis [28]. This is a plausible mechanism for the doxorubicin-mediated disruption of lipolysis, but more studies are necessary.

In contrast, these conditions are antagonistic with regard to lipolysis. In cachexia, the reduction in adipose tissue is a consequence of elevated lipolysis (and HSL activity) [10], and a deficiency in ATGL or HSL in tumor-bearing mice was shown to prevent lipolysis, as well as adipose tissue and skeletal muscle wasting, thus increasing the survival rates in those modified mice [43]. These results indicate that a higher rate of lipolysis is necessary for cachexia development [43].

Taken together, our results demonstrate that doxorubicin treatment strongly affects adipose tissue homeostasis. Both the endocrine and metabolic functions of adipose tissues were impaired in response to doxorubicin. This dysfunction might have been caused by an increase in adipocyte death. Adipose tissues from humans undergoing chemotherapy treatment exhibited a similar phenomenon described as “fat necrosis” [44]. Fat necrosis is the result of adipose tissue death in response to disease, injury, or pathologic conditions.

In conclusion, many side effects of doxorubicin chemotherapy treatment might be induced by the effects of this agent on WAT. Side effects, such as endocrine and metabolic changes, lead to a deep reduction in the quality of life of patients undergoing treatment. Therefore, our results represent an important contribution to an understanding of the side effects observed in patients undergoing chemotherapy and can facilitate the search for alternative treatments that promote a better quality of life.

## Supporting Information

S1 Fig CT mean of constitutive. Gene expression of ribosomal protein L19 (*Rpl19*) at 24 hours, 96 hours and 12 days after induction of cell differentiation in 3T3L1 cells treated with 1uM of doxorubicin. The gene expression was evaluated by real time PCR. Data are mean ± standard deviation of 4 to 6 experiments. Two way ANOVA followed by Bonferroni post test.
(TIFF)

S2 Fig RNA analysis by agarose gel electrophoresis. mRNA analysis by 2% agarose gel electrophoresis visualized by ethidium bromide staining of 3T3L1 cells treated with 1uM of doxorubicin at 24 hours, after induction of cell differentiation. Figure represents 4 experiments per group.
(TIFF)
